# The influence of social relationships and activities on the health of adults with obesity: A qualitative study

**DOI:** 10.1111/hex.13540

**Published:** 2022-06-24

**Authors:** Nestor Serrano‐Fuentes, Anne Rogers, Mari Carmen Portillo

**Affiliations:** ^1^ NIHR ARC Wessex, School of Health Sciences, Faculty of Environmental and Life Sciences University of Southampton Hampshire UK; ^2^ School of Health Sciences, Faculty of Environmental and Life Sciences University of Southampton Hampshire UK

**Keywords:** lived experience, obesity, overweight, qualitative research, social networks, social relationships

## Abstract

**Background:**

Obesity in adults is a leading health challenge that causes millions of deaths worldwide and represents a risk factor for developing long‐term conditions. Social relationships are one of the multiple drivers shaping obesity and obesity‐related practices. However, there is still little evidence as to the processes by which relationships influence the adoption of positive and negative obesity health‐related practices—eating, physical activity and alcohol intake. This study aims first to identify the types of relationships relevant to the adoption of practices in adults with obesity and, second, to explore the type of activities these relationships engage with or promote to produce those practices and their potential health consequences.

**Methods:**

Nineteen adults who have or had a history of obesity living in the United Kingdom were interviewed between May 2020 and March 2021. Experiences were explored through semi‐structured interviews and network mapping via videoconferencing. Data were analysed using a hermeneutic phenomenology informed thematic analysis.

**Results:**

Three main themes were identified: (1) everyday familial routines matter, (2) chasing healthier lifestyles: comparing, modelling and connecting emotionally with friends and (3) healthcare professionals as negative influencers.

**Discussion and Conclusions:**

Findings show how different types of relationships might shape the risk of developing and losing weight. They uncover the power of informal networks (family and friends) and highlight the potentially negative impact of formal ones (healthcare professionals). Our exploration could add to arguments about the need for stakeholders confronting obesity to be aware of the relevance of everyday social relationships in health and well‐being strategies for tackling the issue, in creating collective and individual person‐centred long‐term sustainable actions.

**Patient and Public Contribution:**

Feedback on the tone/content of the interview questions was provided by the two first participants. The results were checked and received feedback from one of the interviewees.

## 1 INTRODUCTION

Obesity is defined as excessive fat accumulation that presents a health risk. From a clinical perspective, for most adults having a body mass index (BMI) greater than or equal to 30 means they have obesity.^1^ It is the fifth leading risk factor for global deaths and represents a significant risk factor for developing a range of long‐term conditions (LTCs), including coronary heart disease, hypertension, type 2 diabetes and joint and muscular disorders, among others.^1^ Currently, 63% of adults in England are above a healthy weight, and of these, 28% live with obesity.^2^ Tackling obesity rates is a health challenge that the United Kingdom has faced during the last decades, with little success.^3^, ^4^, ^5^ Evidence points to its multiple causes, complexity^6^ and the need to extend understanding beyond individuals, including how physical and social obesogenic environments promote obesity risk factors, such as overeating, physical inactivity and energy imbalance. The relevance of physical, economic, policy and sociocultural conditions^7^, ^8^ extends to that of network relationships relevant to the spread and reduction of obesity through social ties, particularly close ties of friends and spouses.^9^ Social networks refer to the structure and function of a person's social relationships and the nature of the ties that connect them. Exploring further the value social networks might play in shaping different health practices has been suggested by Lakerveld et al.,^10^ particularly in understanding the role and meaning of relationships.

Traditionally, social relationships in health have been studied using social network analysis. This method analyses structures by reducing relationships to numbers and bracketing off part of interagency and contextual richness of network interaction.^11^, ^12^ In obesity, the study of the role of relationships has focused on analysing the phenomenon of spread quantitatively, looking at the attributes of people and the risk of developing excess weight through smaller clusters.^9^, ^13^, ^14^, ^15^, ^16^ Powell et al.^17^ attempt to identify different processes through which social ties affect weight influencing behaviours, body size ideals and norms, potentially describing why and how excess weight clusters within networks. In a critical interpretative synthesis review, the content of the relationships and how these interact with environmental and individual determinants to influence the adoption of obesity‐related behaviours^18^ suggests an absence of understanding of the processes by which relationships positively and negatively influence the health of adults with obesity. This study introduces an empirical qualitative inquiry insight to know more about what and how social relationships might influence the adoption of positive and negative obesity health practices related to eating, physical activity and alcohol intake in terms of what respondents narrate about their involvement. Thus, the aims are first to identify the types of relationships relevant to the adoption of practices in adults with obesity and, second, to explore the type of activities these relationships engage with or promote to produce those practices and their potential health consequences.

## 2 MATERIALS AND METHODS

### 2.1 Design

A constructivist epistemology hermeneutic phenomenology was used to inform this study of the lived experience^19^ of how social relationships influence the health of adults with obesity. The researchers' assumptions were not bracketed or set aside but were embedded and essential to the interpretive process.^20^ We used this methodology as part of a qualitative social network approach^21^ in which we wanted to focus on the meaning and narratives of social ties and less on their structure.

Ethical approval to conduct the research was obtained from the Faculty Ethics Committee under the reference ERGO 55638.

### 2.2 Participants

A purposeful sample was recruited since it identifies and selects individuals or groups knowledgeable about or experienced a specific phenomenon,^22^ in this case, those living with obesity in the United Kingdom. This purposeful sampling strategy involved snowballing. Participants were considered eligible for inclusion in the study if they had a current or a history of BMI ≥ 30 kg/m^2^, lived in the United Kingdom, were able to communicate and understand English, had a device with Internet connection and microphone (e.g., mobile phone, tablet, computer) and an email address. They were approached in community settings and via social media (Twitter, Facebook and LinkedIn). Researchers utilized recruitments posters in the Hampshire area (including the Isle of Wight), online advertisements, sent letters of invitation to local weight‐management groups and LTCs‐related charities (asking for their managers' approval when required) from different areas of England, Scotland, Wales and Northern Ireland and contacted with advocacy individuals dedicated to empowering people affected by obesity (via Twitter or email). Managers of organizations in this study were considered those individuals that held leadership roles and could allow access to people who were part of the different organizations and community groups.

### 2.3 Data collection

Individual semi‐structured interviews with open‐ended questions were conducted between May 2020 and March 2021. These types of interviews focus on a specific subject and allow flexibility to capture unpredictable findings.^23^ The participants were interviewed for between 30 and 120 min. The researchers considered the stigma that accompanied this topic and attempted to design an adequate interview schedule where language mattered.^24^ The questions regarding social relationships were designed based on our previous literature review.^18^ Feedback regarding the type and tone of the questions was provided by the qualitative research group members of the authors' affiliation and the two first interviewees (Participants 1 and 2).

**Figure 1:**
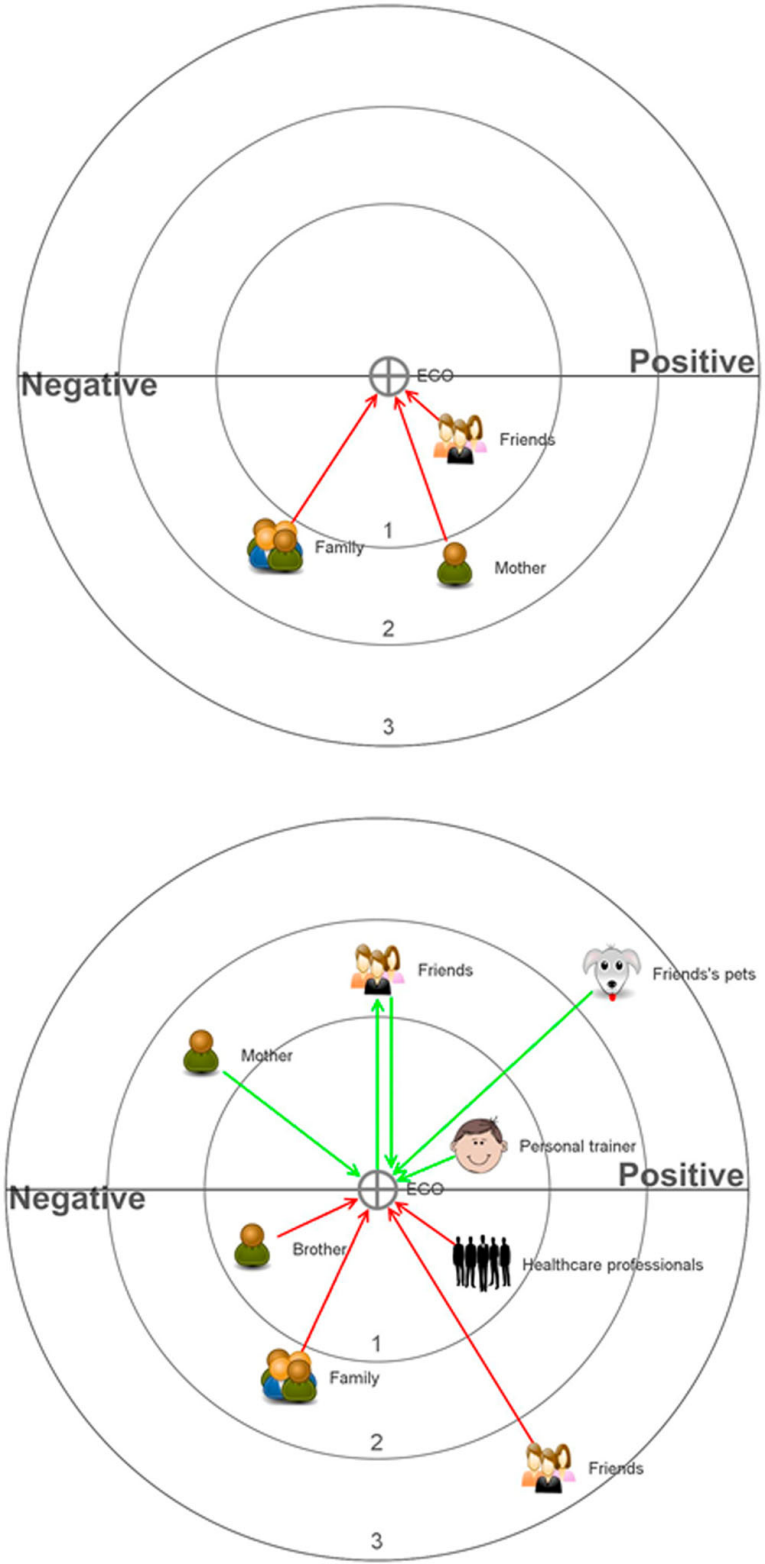
Participant's 1 networks in two different periods (past, top diagram and present, bottom diagram). Red arrows are negative relationships, and green arrows are positive. Those relationships closest to the central circle are the most important.

A personal network sociogram was incorporated to support the identification of social relationships to help generate rich data^25^ by producing reflections about how these relationships influenced participants' health. This sociogram consisted of three concentric circles.^26^ The interviewer started the interview by asking, ‘Who do you think is most important to you in influencing your health behaviours (eating, physical activity and alcohol intake), positively and negatively’? Thus, the interviewees could place proposed network members in either the central circle considered essential, the middle circle considered important but not as relevant as the central circle or the outer circle, considered necessary but not as important as the other two circles. Two diagrams were collected per person representing a moment in the past (when they started having obesity) and the present. While filling in the diagrams out loud, participants were asked to explain why they chose the people they had placed within the circles and how they had influenced their health. An example diagram can be seen in Figure 1. Additionally, a sociodemographic questionnaire was used to register different attributes and establish relationships with the individuals' conception of health experiences and opinions.

Due to the coronavirus pandemic, all data collection was conducted online, specifically via email and videoconferencing. Emails were used to contact the participants and send information about the study (e.g., participant information sheet). SafeSend service (software hosted by the research institution) was utilized to transfer research and confidential data across the network securely encrypted. Consent was gained from all participants before the initial interviews, which were conducted by videoconferencing. This method was chosen in comparison with others (e.g., telephone) since it provides a more personable approach,^27^ something that was found relevant when discussing this sensitive topic. Phone calls were considered if there were any connection or technological problems or at the interviewee's request. Thus, 18 interviews were carried out via videoconference and one by telephone. N. S. F. conducted all the interviews and recorded using a Dictaphone (Olympus WS‐853). Researchers had no therapeutic relationship with participants.

### 2.4 Data analysis

The interviews were transcribed verbatim, 10 of them by the first author (N. S. F.) and the rest by a professional transcriber. Template thematic analysis was utilized to identify, analyse and report repeated patterns across data.^28^ Data analysis followed a deductive and inductive analysis process. Deductive in the sense that themes can be developed early on or even before the analysis.^29^ Inductive in the sense that new themes and codes are created through the analytic process. Template thematic analysis was chosen in comparison with other types of thematic analysis since it allows a‐priori themes to be utilized to create an initial version of the coding template.^30^ The different phases involved in the analysis can be seen in detail in Material S1.

To increase the validity of the analysis, the primary author conducted a debriefing with the rest of the authors (A. R. and M. C. P.). Two tools were used to ensure the quality of the results: the COREQ checklist^31^ and the tool for evaluating thematic analysis manuscripts developed by Braun and Clark.^29^ The different domains with our answers can be seen in Materials S2. Also, Participant 1 was contacted to verify the interpretation of the findings (no issues were raised). After the 17th interview, no new codes and themes were generated from the narratives. Thus, the authors concluded that the data analysis had reached a saturation point (data saturation).^32^ However, two more interested participants were interviewed to ensure and confirm that there were no new emerging codes and themes.

All the real names were coded into pseudonyms to ensure anonymity. Regarding software, NVIVO (version 1.2) supported the labelling and organization of themes.

## 3 RESULTS

Nineteen adults (13 women and 6 men) who have or used to have obesity (BMI ≥ 30)^1^ were interviewed. Table 1 summarizes the main attributes of each participant and Table 2 shows a list of all themes, subthemes, codes and their frequencies.

**Table 1:** Participants' sociodemographic characteristics

Participants	Age	Current BMI	Long‐term conditions	Civil status
1. Female	25–29	36	Underactive thyroid	Single
2. Female	40–44	36	Irritable bowel syndrome, arthritis, fibromyalgia	Married/live‐in partner
3. Female	40–44	34	Mild asthma, back pain	Divorced
4. Female	25–29	35	Asthma, polycystic ovarian syndrome	Married/live‐in partner
5. Female	45–49	34	Nil	Separated
6. Male	30–34	33	Nil	Single
7. Female	25–29	25	Nil	Single
8. Female	25–29	33	Nil	Single
9. Female	55–59	30	Barrett's oesophagus	Separated
10. Female	30–34	29	Nil	Single
11. Male	40‐44	28	Nil	Single
12. Male	65–69	35	Nil	Married/live‐in partner
13. Male	45–49	29	Depression, reactive arthritis	Married/live‐in partner
14. Male	35–39	49	Psoriasis	Married/live‐in partner
15. Female	50–54	27	Asthma	Married/live‐in partner
16. Female	55–59	39	Nil	Married/live‐in partner
17. Male	35–39	24	Nil	Single
18. Female	25–29	33	Underactive thyroid	Married/live‐in partner
19. Female	18–24	40	Nil	Single

Abbreviation: BMI, body mass index.

**Table 2:** Themes, subthemes and codes

**1. Everyday familial routines matter (18/124)** 1.1 Activities with positive effects on health (18/72) 1.1.1 Family (in general) (3/5) 1.1.1.1 Being a role model (2/2) 1.1.1.2 Conducting activities together (1/1) 1.1.1.3 Counselling (1/2) 1.1.2 Parents (13/20) 1.1.2.1 Being a role model (1/1) 1.1.2.2 Physical loss (2/3) 1.1.2.3 Peer pressure (3/4) 1.1.2.4 Conducting activities together (1/1) 1.1.2.5 Counselling (3/3) 1.1.2.6 Emotional support (3/3) 1.1.2.7 Education in cooking and eating healthy (4/5) 1.1.3 Pets (5/7) 1.1.3.1 Providing physical exercise (5/7) 1.1.4 Partners (12/23) 1.1.4.1 Cooking and shopping healthy food (2/7) 1.1.4.2 Counselling (3/3) 1.1.4.3 Emotional support (4/4) 1.1.4.4 Sharing lifestyle goals and making joint efforts (3/3) 1.1.4.5 Comparing (4/4) 1.1.4.6 Being a role model (2/2) 1.1.5 Children (6/12) 1.1.5.1 Having similar characteristics (1/1) 1.1.5.2 Emotional support (2/2) 1.1.5.3 Cooking and shopping healthy food (1/1) 1.1.5.4 Education in eating healthy (1/3) 1.1.5.5 Peer pressure (4/5) 1.1.6 Grandparents (2/2) 1.1.6.1 Comparing (1/1) 1.1.6.2 Emotional support (1/1) 1.1.7 Siblings (2/2) 1.1.7.1 Counselling (2/2) 1.1.8 Aunts and uncles (1/1) 1.1.8.1 Emotional support (1/1) 1.2 Activities with negative effects on health (16/52) 1.2.1 Family (in general) (5/9) 1.2.1.1 Having similar characteristics (2/3) 1.2.1.2 Peer pressure (1/2) 1.2.1.3 Growing up seeing and modelling bad practices (1/1) 1.2.1.4 Judging, labelling and commenting (1/1) 1.2.1.5 Conducting activities together (1/2) 1.2.2 Parents (12/25) 1.2.2.1 Providing an excess of control (5/7) 1.2.2.2 Conducting activities together (1/1) 1.2.2.3 Cooking and shopping (7/13) 1.2.2.4 Growing up seeing bad practices (4/4) 1.2.3 Pets (1/1) 1.2.3.1 Encouraging physical activity (1/1) 1.2.4 Partners (4/8) 1.2.4.1 Having similar characteristics (1/1) 1.2.4.2 Peer pressure (2/2) 1.2.4.3 Comparing (1/1) 1.2.4.4 Conducting activities together (3/3) 1.2.4.5 Eating and shopping (1/1)	1.2.5 Children (1/1) 1.2.5.1 Education in cooking healthy (1/1) 1.2.6 Grandparents (3/4) 1.2.7.1 Peer pressure (1/1) 1.2.7.2 Cooking and providing excess amount of food (2/3) 1.2.7 Siblings (4/4) 1.2.8.1 Peer pressure (1/1) 1.2.8.2 Physical loss (1/1) 1.2.8.3 Judging, labelling and commenting (2/2) **2. Chasing healthier lifestyles and modelling and connecting emotionally with friends (18/87)** 2.1 Activities with positive effects on health (18/68) 2.1.1 Close friends (15/41) 2.1.1.1 Having similar characteristics (3/5) 2.1.1.2 Modelling (1/2) 2.1.1.3 Comparing (3/3) 2.1.1.4 Cooking and shopping healthy food (1/1) 2.1.1.5 Conducting activities together (6/9) 2.1.1.6 Counselling (5/6) 2.1.1.7 Emotional support (11/15) 2.1.2 Community friends (gym and weight management groups) (9/23) 2.1.2.1 Leadership and counselling (3/5) 2.1.2.2 Sharing agendas and aims (3/3) 2.1.2.3 Conducting activities together (1/1) 2.1.2.4 Emotional support (5/9) 2.1.2.5 Comparing (1/1) 2.1.2.6 Modelling (1/2) 2.1.2.7 Peer pressure (2/2) 2.1.3 Colleagues at work (2/4) 2.1.3.1 Emotional support (1/1) 2.1.3.2 Conducting activities together (1/1) 2.1.3.3 Comparing (1/2) 2.2 Activities with negative effects on health (12/19) 2.2.1 Close friends (10/14) 2.2.1.1 Comparing (1/1) 2.2.1.2 Cooking and shopping healthy food (1/1) 2.2.1.3 Conducting activities together (5/5) 2.2.1.4 Counselling and mentoring (1/1) 2.2.1.5 Peer pressure (5/6) 2.2.2 Community friends (gym and weight management groups) (3/5) 2.2.2.1 Comparing (2/3) 2.2.2.2 Counselling (2/2) **3. Healthcare professionals as negative influencers (11/20)** 3.1 Activities with positive effects on health (2/3) 3.1.1 Emotional support (1/1) 3.1.2 Counselling (2/2) 3.2 Activities with negative effects on health (10/17) 3.2.1 Counselling, patronising and not providing person‐centred plans (7/12) 3.2.2 Communication, lack of a sensitive approach (4/5)

*Note*: Two numbers have been added per data item. The first number represents the number of interviews where the item appears. The second one represents the number of times the item has been identified in all the interviews.

In the main, three types of social relationships were identified through the narratives and network diagrams: family members, friends and healthcare professionals. The activities by which these relationships could influence participants' health are explained in detail below. The interviewees reported potential health consequences related to mental health and the adoption of different health practices regarding eating, physical activity and alcohol intake. Also, broader social and economic contexts within which these interactions were situated could be identified as part of the narratives.

Three main relational themes were identified: (1) everyday familial routines matter, (2) chasing healthier lifestyles: comparing, modelling and connecting emotionally with friends and (3) health care professionals as negative influencers. A complete list of all the codes can be seen in the final version of the coding template in Material S1.

### 3.1 Everyday familial routines matter

Participants reported histories of influences stemming from interactions in domestic settings over time, starting from childhood to the present. Family members were the most mentioned people in terms of influence on participants' health. Eighteen participants addressed the importance of their families in everyday routines, which could induce different health‐related responses from participants.

#### 3.1.1 Activities with positive effects on health

Past interactions with family members during childhood have salient current meaning and positive effects on some participants' lives. For example, mothers providing education on cooking and eating healthier was reported as having effects on participants' current relationship with food, and knowing what they should eat to prevent gaining weight.

*I think a positive influence of my family background is that I was taught cooking. So from a boy I was able to cook and that makes all the difference*. Participant 13

*For me my relationship with food is probably completely different and I think also again for my upbringing my mum was very much she was really good with making sure we ate well*. Participant 18

Sharing contemporary lifestyle goals and making joint efforts with partners to achieve those goals were reported several times during the interviews. For instance, participant 10 explained the interaction with her boyfriend to motivate themselves, exercising and preparing healthier food together:

*My boyfriend influences me a lot: we go to the gym together, we cook together, this week we talk about cooking healthier food, we go running 3 or 4 times a week. We are always saying to each other, come on, now we are going to run because we are relaxing*. Participant 10

Children were identified as ties that influenced. For example, some parents pointed out their honesty to comment on things and exert pressure as reminders to be aware of what they needed to improve. These positive interactions could work in both directions. Thus, Participant 5 highlighted how her children increased her awareness of what she was doing wrong, and, consequently, this could enhance her to improve her shopping, cooking and teaching practices towards them.

*They are both teenagers, they started giving back so they would tell me we really need to have more vegetables mummy or why are we having chips again…, it's started working both ways because when you are a parent and they are little you take decisions for them and you teach them, etc*. Participant 5

Pets were found to have a role in providing opportunities for physical exercise for their owners. For some, dogs encouraged daily physical activity and, also, walking the dog with friends who also owned pets provided a supportive context to connect with others and conduct exercise together.

*The last 18 months I've done my main exercise had been walking the dog, but not great distances. The Labrador I used to love going 4‐5 miles*. Participant 12

*I'm not close to the forest but it's only a car journey away so I've got friends that have animals and stuff as well so we've been on dog walks together*. Participant 4

#### 3.1.2 Activities with negative effects on health

Several participants reported growing up seeing and modelling bad eating habits from members of their families. For example, participant 10 highlighted that gaining weight and developing a ‘strange’ relationship with food could have its origin during childhood since her father had already bad habits around food:

*My father's way of eating is brutal. He eats fat, sugar, he eats very badly; eat everything that should not be eaten; vegetables, once a month. Well, I imagine that during my childhood; all that has affected me a lot, developing a relationship with food that is a bit strange*. Participant 10

Also, parents could provide an excess of control rather than adequate emotional support. Some participants described their parents as monitoring and using normative judgemental attitudes, questioning interviewees' efforts and provoking tensions within the relationship. Participant 13 mentioned these issues over the years:

*My parents, 'cause they are negative. There's not so much they do anything now, but there's been an historical perspective on it, which is that my parents were very when I was growing up, were very invasive in terms of my body shape*. Participant 13

Conducting everyday life with family members in tandem could be a potential risk of gaining weight. For example, Participant 13 explained the relevance of sharing drinking and sedentary practices with his partner over the years and recognized the importance of conducting together other activities to work towards opposite effects on health:

*"Most important person to me unquestionably has to be my husband. So basically we've been together for 24 years now, and on our health and well‐being lifestyles are very pretty much together, always hard, isn't it? With diet because when you have somebody who is there to share a bottle of wine or you know or to watch a film instead of doing some exercise either you sort of bad for each other, and so we tend to put on weight together at the same time, and the health deteriorates over time*. Participant 13

Social relationships occur within specific social contexts, and broader social and economic factors could shape some activities within these interactions. For example, family economic conditions and food prices could have modified the type of food that some parents bought and cooked for their children. Participant 1 commented on the difficulties when there was insufficient income at home during her childhood, which could influence the type of food they were having at home:

*My mum tried her best but that was, obviously quick easy food was a bit cheaper as well and obviously being a single parent she got what she could afford*. Participant 1

Another broad factor was the restriction and changes to everyday practices in response to COVID‐19 isolation policies, which could have modified how families bought and cooked food for the worse:

*With this pandemic, there were a few occasions when I actually served to my children a ready‐made lasagne, heavily reduced. When I did it the second time, they said oh my gosh mummy are we going to eat ready meals from now on*. Participant 5

*The pandemic was difficult, shopping was horrible, between queues, the first days the fact that there was nothing on the shelves. We believed in comfort eating*. Participant 14

### 3.2 Chasing healthier lifestyles: Comparing, modelling and connecting emotionally with friends

The influence of friends was the second most mentioned type of relationship among the participants, and several activities, most of them with a potentially positive impact on health. Comparing with friends who had already healthy lifestyles and modelling their practices was identified. For example, Participant 8 started hanging out with a healthy friend to move from inactivity to exercise. Also, Participant 12 was suggested for trying a new diet that caused his friends to lose weight:

*A very close friend of mine promoted healthy food a lot, we go to the gym and for walks together*. Participant 8

*A friend of mine, last September and his wife. He sent a picture of him and his wife. And he said, what do you think? We've lost about three stone and we're going to a wedding and I've to go and get a new suit because this one don't fit anymore and he was like holding these trouser waist out a massive gap. Have you done that? They just followed a diet called Asansi, and he said you wanna give it a go? So I gave it a go for two*. Participant 12

Engaging with different community resources, such as gyms and weight management groups, provides opportunities to build relevant friendships, which could influence in different ways. For example, exercising with other gym members was relevant to establishing a supportive psychological network and maintaining habits. This was explained by Participant 6, who used to have obesity and started a gym journey to lose weight and keep fit:

*Then because you go to the gym, because you do exercise, because you know how it feels to be overweight in the past, then you start hanging out with friends or making new friends that help you in the support not just psychological support but also when it comes to going to the gym or doing any sport or any other activity but that you like, that you consider important for you in your life*. Participant 6

Leadership and support provided by personal trainers enhanced behaviour change and the maintenance of habitus during a fitness journey. Participant 1 highlighted a person‐centred education and the emotional support provided by her personal trainer (PT), whom she already considered a friend.

*So I started with my PT about a year ago, over a year ago, on my fitness journey, whatever you want to call it, and he has taught me a lot, he's taught me about myself, he's taught me about food, he's taught me about my relationship with food and he's also taught me about my exercise. So actually at the moment in terms of actual aspects of health he has probably been one of my biggest positive motivators at the moment. He's just helped me with my health behaviours and the fact that food and exercise can be enjoyable. He's become a friend probably at the end of all of this as well which is really good*. Participant 1

Friends from weight management groups shared agendas and aims, such as chasing specific lifestyle goals. Living similar experiences allowed them to connect emotionally with other participants. Also, they could compare with each other and be a source of motivation to change practices. This was explained by Participant 15:

*My running clubs and my bootcamp class, they influenced me the most, more than my family, because they are encouraging and they are friendly and competitive. So in terms of my weight loss journey and my weight now, I think the people that are most important and influences me the most are certainly my friends that I've met through the clubs, running and bootcamp club…But I think that other magic of slimming world apart from the good food, is it's a group thing, this incredible community spirit of people who are going through the same stuff, meet up once a week for an hour and have a chat about what's difficult for them and how to change their lives*. Participant 15

Friends who did not experience similar life circumstances were found to be crucial in providing emotional support. Thus, trust and the degree of intimacy between close friends, respect and understanding were reported. For example, Participant 9 stated being able to talk to any of his closest friends about weight concerns.

*A few of my friends I think I would put there. And could have quite frank conversations about how I'm feeling, how they are, if you've got any weight problems, yes to be able to talk to them more*. Participant 9

On a few occasions, friends were found to influence negatively. Thus, most adverse health practices from their influence happened when modelling them in social gatherings and meetings. These involved eating worse and drinking above the recommended amounts of alcohol in a company. Participants 7 and 11 identified conducting certain activities and modelling what others did to facilitate social interactions.

*You go out with your friends, you have drinks, you go out for food, it's all just, if your friends smoke you are more likely to smoke, it's hard to avoid things that are not necessarily good for you if your peer social group do it as well*. Participant 7

*Where before you end up just getting flush and drunk because it's available, it's free, it's being sociable if you know what I mean*. Participant 11

### 3.3 Healthcare professionals as negative influencers

Different types of healthcare professionals, mainly General Practitioners (GPs) were identified to have a negative impact. Although the interviewees had less contact with them, they were considered in the main to be unhelpful. Ten participants recognized them as negative influencers to managing obesity by increasing the stigma, not exploring each case in‐depth or not providing the right tools to improve physical activity levels or diet. For example, Participant 1 pointed out that healthcare workers were patronizing and did not provide person‐centred individualized plans over time:

*I've found them to be quite patronising over the years because I've gone to them a few times and obviously being like I've got these issues and they're like well you need to do this, this and this, you need to change this and it's like OK how are you going to help me do that. And they're like oh, that's not an excuse my daughter does this, and it's like well but your daughter is very different to me, you are not being individualised of me*. Participant 1

Additionally, other participants mentioned the lack of a sensitive approach to the language. For example, Participants 2 and 7 highlighted the repetitive use of certain words such as ‘obese’ or ‘fat’, words they do not find very supportive and make them feel upset.

*So every time I go and see my GP he tells me I'm too fat, he tells me I'm morbidly obese, which is not very helpful*. Participant 2

*And I know when I was obese and I went to the doctors it just made me more defiant, they were like you are obese and I was like no I'm not. It's just not a very kind way of dealing with it*. Participant 7

Participant 14 even stated that his GP obviated that he was taking some medication (for another condition) whose side effects were weight gain. However, the clinician was justifying his obesity with a bad diet:

*I went to the GP and he told me you have fitting yourself into obesity. That after having been taking, and he knew it, I was taking Mirtazapine for months that make you gain weight. Going to a GP because you are taking depression pills and then he is telling you that you have become obese because of what you have eaten…it does not help your mental health either*. Participant 14

## 4 DISCUSSION

This study moves away from the traditional understanding of networks and obesity, which has focused on studying structures and their spread in clusters of people. Instead, this qualitative inquiry sought to gain an understanding of how and why some social relationships influence the everyday health of adults with obesity. Specifically, our results illuminate the contingent and multifaceted ways in which networks can influence obesity‐related practices in adults related to eating, physical activity and alcohol intake.

The visualization of network diagrams and the narratives show that immediate family members were the most important relationships for participants. The potential impact of the family is not new, and it has been explored before, for instance, in relation to people living with type 2 diabetes.^33^, ^34^ This study adds to existing evidence by identifying and describing positive and negative interactions over time, starting during childhood and continuing today through everyday routines in these domestic settings. From a positive perspective, parents provided education on how to cook and eat healthy during the early stages of some participants' life, which contributed to their current and healthy relationship with food. In addition, elements of mutual influence were found, such as sharing lifestyle goals and making joint efforts with partners to eat healthier and increase physical activity levels and the pressure exerted by children to eat healthier. Consequently, some parents (participants) changed their shopping and cooking practices. Similar to our findings on the positive impact of children, other authors point to the role of children in supporting participants in making healthy shopping choices.^35^ Pets (considering them as part of the family) provided daily exercise to their owners. This supports existing evidence on pets' positive role in supporting people with other long‐term health problems.^36^ From a negative perspective, growing up seeing and modelling bad eating practices from parents, exerting excessive control and pressure by parents, again sharing and conducting, in this case, nonhealthy lifestyles with partners and buying and cooking unhealthy food depending on economic capabilities were reported. In line with the latest, Thayer et al.^37^ pointed out in a recent study on teenagers how a family's financial position influenced what foods their families purchased in stores.

Friends were the second most mentioned type of influential relationship, and their impact was usually positive. The participants identified the role of intimate or closest friends and friends from other meaningful social systems (weak ties).^38^ The latest was friendships built in community settings as part of the participants' lifestyle journey, providing opportunities to access diverse resources.^39^ These results echo more recent evidence, which suggests the relevance of weak ties for the self‐management of other LTCs.^40^, ^41^ Considering the activities within these friendship interactions, comparing with intimate friends who already had healthy lifestyles and modelling their practices were drivers to increasing exercise levels and trying new diets. Also, engaging in gyms and weight management groups (community settings) offered the space to interact with personal trainers, gym and group members (weak ties friends) to whom share similar lifestyle norms, compare, model and find a source of motivation, information and emotional support to change and keep practices over time. This is the only relevant difference between the participants who had lost weight and do not have obesity currently and those with obesity. Thus, most of those who have been successful in their weight loss journey had more varied networks with the inclusion of community interactions. Other studies show how finding people with similar characteristics and lifestyle norms improve people's capacity to improve their mindset and motivate changes.^42^ As with previous research the interviewees reported emotional support to understand and respect new choices from intimate friends.^43^ The only negative influence of friends was noted when eating and drinking were involved in meetings, potentially due to sharing norms (e.g., drinking alcohol when socializing) or modelling others to facilitate social interactions. This supports existing evidence about the impact of friends on social events and gatherings in other countries,^44^ and that individuals eat more when their friends eat more and eat less when their companions eat less.^45^

In addition to the influence of informal networks (family and friends), the participants identified the impact of healthcare professionals, although they were more peripheral in the network diagrams on most occasions. Although this is not a new issue in the literature, the negative influence of healthcare professionals^46^, ^47^, ^48^ (we could name them formal networks) was reported. The interviewees detailed the lack of effective counselling and individualized care plans to change health practices, and the lack of a sensitive approach and harmful language during the communication, such as reiterating the use of ‘obese’ and ‘fat’. Current research by other authors in the United Kingdom explores how healthcare professionals consult with people living with obesity regarding weight loss and identify similar issues.^24^, ^49^, ^50^ For example, the use of a biased, generalized and nonperson‐centred language, conversations of causes of obesity focusing on individuals' management and the lack of a collaborative approach between the professional and the person to identify individual, meaningful goals were highlighted by others and confirmed by us.

Contemporary intervention guidelines^51^ and prevention lifestyle measures mention the benefit of receiving support from different individuals to lose weight and prevent weight regain. Most of this information seems very superficial and does not delve into how our daily and closest relationships can cause positive and negative health changes and the opportunities and the mechanisms by which different people from the community can impact the weight journey. Our results and the way data were collected could be implemented in practice change and reproducing habitus of meaningful practices. Clinicians could use the described inquiry process (interviews and network mappings) to reflect on the impact of individuals' relationships and plan specific person‐centred and long‐term sustainable network interventions to improve individuals' physical and mental well‐being in people with obesity and its associated conditions. Within the planning of actions, patients could tailor individual preferences, deciding what could work for managing their health and well‐being. For example, behavioural treatments for people with obesity could include family as part of changing daily routines involving eating or physical activity. The role of friends as key to designing interventions seems to be warranted given their salience and input on obesity‐related practices. Interventions incorporating close personal contacts, in particular learning skills, have been found to be effective.^52^ Also, it could be useful to increase awareness of local community health resources and how the people who participate in them could influence change practice. Different innovative interventions^53^, ^54^ are being tested, facilitating health services addressing these issues through social prescription. The use of network mapping could be used as a reflective process by the general population. Who is important, whom they should keep, whom they should avoid or whom they should include in their network and how they influence are some prompts that the network mapping could help reflect on.

Furthermore, this study adds evidence to the necessity of changing how obesity is approached in clinical settings. Therefore, it might be necessary to provide further education on weight management, including managing delicate discussions and stressing the multiple and complex underlying causes of obesity. This could avoid scenarios where people with obesity could self‐blame as a result of negative interactions with healthcare professionals. For example, this awareness and actions in clinical practice could be implemented by introducing guidelines created by healthcare professionals, obesity researchers, people living with obesity, carers and other stakeholders who have worked collaboratively to address these issues.

The visualization and the narratives of our participants' network diagrams in different moments in life presented the involvement of varied types of relationships, showing the importance of considering historical life perspectives to understand the process of gaining and losing weight and the role of networks, understanding them as dynamic entities rather than static.^55^ For future research, the use of biographic narratives (to understand more in‐depth the content of meaningful relationships over time) with the exploration of the structure of social relationships using network metrics could provide an upgraded tool to explore their impact on health over time.

Also, we encourage other researchers to use qualitative techniques to explore in‐depth private accounts about living with obesity to continue gaining a necessary and still sparse understanding of its multiple causes and the meaning of living with it. This could be used as part of participatory action research in which researchers and the affected populations work together as agents of change to generate strategies to tackle obesity and its related health and social issues. Involving their voices to develop new policies must be a priority to address the current psychology and aetiology of obesity and ensure that they receive adequate quality care with effective and person‐centred interventions. Also, their empowerment and taking roles in patient and public advocacy could contribute to ending still some crucial barriers, such as weight stigma and discrimination.

### 4.1 Study strengths and limitations

One of the strengths of this study was the use of network mapping to identify members of participants' social networks and reflect on how these influenced their health behaviours. Furthermore, the narratives not only showed the enmeshed and collective nature of the negative influences to adopt unhealthy practices but also how relationships could be a solution to conduct healthier lifestyles and reverse the effects of obesogenic environments from a microperspective.

On the other hand, this study and its development context show challenges and limitations. The coronavirus pandemic, the lockdowns and the fact that this topic encompasses significant social stigma challenged the recruitment process and the possibility to attract more interested potential participants. Forty‐four local and national weight management groups and obesity and LTCs charities were contacted with no success. The majority did not reply to the requests (a follow‐up email or message was sent to each potential participant and group if there was no initial response). A few just declined the potential participation without providing reasons. Others stated that the lockdown period was not the moment to conduct this type of research. Also, we could not obtain a varied sample of participants (e.g., different sociodemographic characteristics) and we did not explore the relationship between the individuals' attributes and their attached health opinions. Therefore, the new understanding of relationships' influences must be considered cautiously and not establish generalizations since they might not apply to people with other sociodemographic characteristics. Another potential limitation might be the accuracy of the network diagrams. The original idea was to collect the network data in person with the participant. However, the pandemic changed the way data were collected. Although we sent the concentric circles with some instructions to think about and draw their networks several days before the interview, some people did not conduct the exercise. This could lead to exaggerating some relationships and omitting others that may be important to their health^56^ when interviewing online due to the limited availability in time.

## 5 CONCLUSIONS

This qualitative approach offers a new way to explore what and how social relationships influence the health of adults with obesity and thus point to the content of intervention that foregrounds relational resources in changing obesity‐related practices. Specifically, it identifies different types of relationships, activities within these interactions and specific contexts that could induce positive and negative health outcomes. As part of our results, we raise awareness of and reflection on the relevance of informal relationships (family and friends) and the negative impact of healthcare professionals (as formal relationships) to manage obesity and its wider‐related health issues. These relationships can shape the risk of gaining and losing weight and impact mental health through the different activities we have unravelled. Our exploration could make the general population and stakeholders aware of the power of social relationships in health and well‐being, suggesting their relevance to creating individual person‐centred and long‐term sustainable actions.

## Supporting information

Supplementary information.

Supplementary information.

## Data Availability

The data that supports the findings of this study are available in the supplementary material of this article.
