# Supplementary material for: The influence of social relationships and activities on the health of adults with obesity: A qualitative study
Source: Health Expect. 2022 Jun 24;25(4):1892–903. doi: 10.1111/hex.13540 (PMC9327877; doi:10.1111/hex.13540)
Supplement: Supplementary file 1 — Supplementary information. [file HEX-25--s002.docx]

**Supplementary material 1. Data analysis in detail using template thematic analysis**

**BEFORE THE ANALYSIS: TRANSCRIPTION**

The interviews were transcribed verbatim, ten of them by the first author (NSF) and the rest by a professional transcriber. The support from the latest was needed since the sound quality was not ideal in some of the interviews conducted online. All the transcripts were checked for errors by listening back to the audio recordings and reading the transcripts at the same time. Small notes were specified in the transcripts to identify nonverbal communication, long pauses, or some change in the tone of voice. This could support the coding process.

For this study, template analysis was chosen. Thus, a list (template) of hierarchical codes (themes and codes) representing areas of knowledge identified in their textual data was produced. Five phases were involved in this research:

**STAGE 1: FAMILIARISATION**

Familiarisation with the data, becoming immersed with the content. The main author of the research listened back to the audios and read a couple of times each interview transcript to have a ‘whole picture’ of the entire data set.

**STAGE 2: CREATION OF AN A-PRIORI TEMPLATE**

An *a priori* hierarchical template with themes (broader patterns of meanings) and codes (lower-order codes) was created. These were modified through inductive data engagement. The *a priori* template was identified through previous relevant research. Thus, themes and codes were developed from our previous critical interpretative synthesis review:

*Serrano Fuentes N, Rogers A, Portillo MC. Social network influences and the adoption of obesity-related behaviours in adults: A critical interpretative synthesis review. BMC public health. 2019 Dec;19(1):1-20.*

A priori template

| **Types of ties of social networks for the adoption of obesity-related behaviours**  Meso-level ties  Sport contacts  Healthcare professionals  Neighbours  Community organizations and community (others)  Micro-level ties  Family  Friends  Housekeeping  Co-workers  Pets  **Types of properties of social networks for the adoption of obesity-related behaviours**  Tie strength  Degree  Size of network  Degree of separation  **Meso-micro network processes for the adoption of obesity-related behaviours**  Meso-level processes  Social support (peer and group)  Homophily  Social pressure  Natural communication  Social modelling  Diffusion  Micro-level processes  Social modelling  Social comparison  Social pressure  Social support (peer and group)  Homophily  Natural communication  Isolation  **Contextual and individual factors for the adoption of obesity-related behaviours**  Micro-level factors  Environmental factors  Built environment  Community resources  Weather  Socio-cultural factors  Social events  Social norms  Micro-level factors  Environmental factors  Group atmosphere  Psychosocial factors  Critical moments  Motivations  Self-efficacy  Knowledge  Personal attitude  Socio-cultural factors  Social norms  Competing demands  Social events  Sociodemographic factors  Socio-economic status  Job  Age  Gender  Transport  Education  Clinical factors  Medical conditions |
| --- |

**STAGE 3: CODING**

The initial template was applied to start coding (generating labels) the data set, highlighting parts of the text that contributed towards the research aim.

The first author underlined interesting text segments (parts of sentences, whole sentences or even paragraphs) and used the right-hand margin to specify codes or labels. Notes and ideas were written in a printed version of the transcripts. Below, an excerpt of open coding from the first attempt of coding is presented. In this example, Participant 3 mentioned that some work colleagues were living the same situation as her. Thus, they were working out together how they could lose some weight and enhance motivation by creating a competitive atmosphere. On the right part of the screen, some codes were highlighted. In that case, we created the codes *mindset*, *co-workers* and *collective efficacy* for that text fragment.


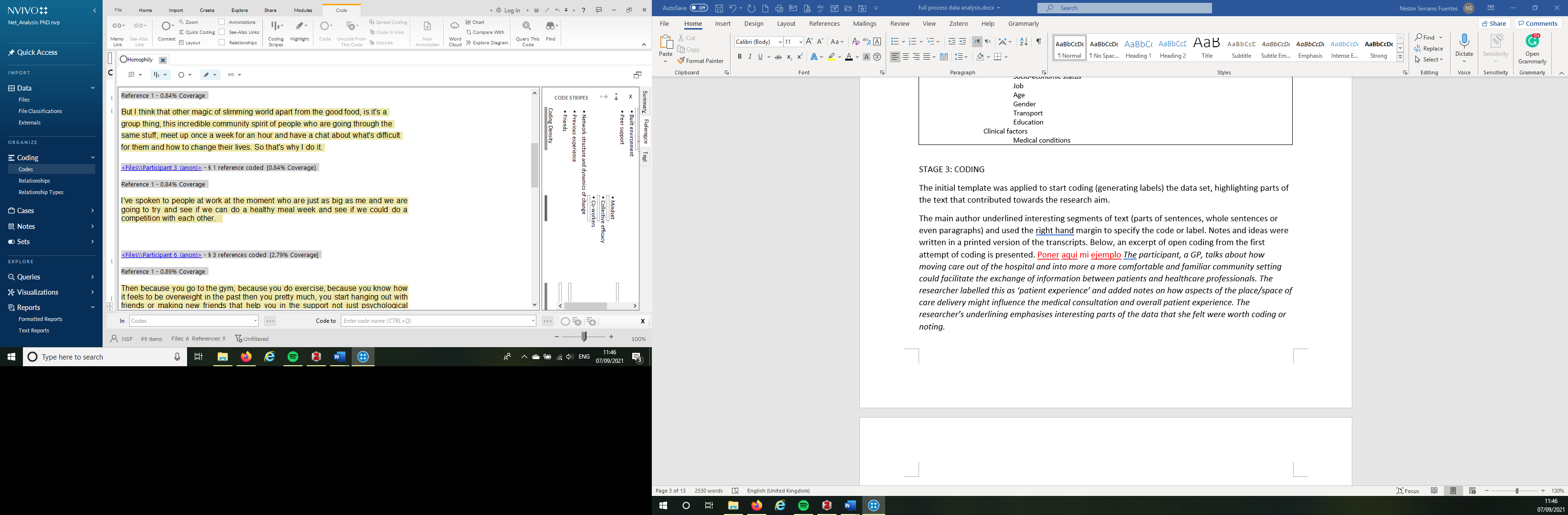


Screenshot from NVIVO when doing the analysis

The initial template was continuously modified during the analysis process. Below, different versions are presented, and changes are explained.

TEMPLATE VERSION 1

After the first five interviews, a first review of the data took place, and the first suggestions were made by the rest of the team. Thus, the initial template started to change. New codes were created, and existing ones redefined or even deleted from the template. The themes *importance of networks* and *network structure and dynamics of change* were created. At this stage of the research, we were willing to include all the characteristics related to the structure of social networks (although they were omitted later, as explained below). Also, the levels of analysis (meso and micro) were removed. The different *types of processes* and the *contextual and individual factors* were divided into positive and negative effects.

Template version 1

| **Type of ties**  Online networks  ex-husband  Personal trainer  Pet  Neighbours  Sport contacts  Co-workers  Community group  Boyfriend and girlfriend  Housemates  Friends  Family  Healthcare professionals  **Contextual and individual factors**  Contextual  Negative  Prices  Social norms  Family socioeconomic status  School activities  Bars and pubs  Working environment  Social events  War times  Change country  Coronavirus  Positive  Networks with conditions  New country  Social norms  Built environment  Social media  Community services  Incorporation to work  School starting September  Individual  Positive  Knowledge  Mindset  Age  Motivation  Awareness  Critical moment  Previous experience  Happiness  Negative  Marital status  Socioeconomic status  Work  Genre  Self-management  Mindset  physical appearance  Mental health | **Processes, ties and outcomes**  Negative  Social support  Competing  Diffusion of practices  Isolation  Death  Social modelling  Homophily  Bullying  Socialising  Social comparison  Peer pressure  Peer support  Patronising  Judging  Exercising  Mentoring  Labelling  Advising  Shopping  Collective efficacy  Cooking  Positive  Competing  Social awareness  Homophily  Community support  Social modelling  Peer support  Companion  Social prescription  Caring  Walking  Advice  Emotional  Trust  Mentoring  Collective efficacy  Cooking  Social comparison  Peer pressure  **Importance of networks**  **Network structure and dynamics of change** |
| --- | --- |

TEMPLATE VERSION 2

Although some changes were produced, the template from version 1 to version 2 kept a similar structure.

Type of ties

*Online networks* were removed. At this point, *social media* (contextual factors) was considered to be an intermediary between the different participants. *Influencers, YouTubers (online networks)* were not considered for this study.

The code *family* was divided into more minor codes (members of the family). Thus, *aunts and uncles*, *children*, *siblings*, *grandparents*, *partners*, and *parents* were added.

*Classmates* were added as a code.

Contextual and individual factors

Contextual negative

*Prices* and *family socioeconomic status* were included in the category *Individual-socioeconomic status*. *School activities, war times* and *change country* were deleted as irrelevant. *Bars and pubs* were included in *social events*. *Built environment* was added.

Contextual positive

*Green spaces* was added to *built environment*. *Network conditions* was added into *individual-previous experience* and deleted from *contextual factors*. *New country*, *community services* and *incorporation to work* were deleted as irrelevant.

Individual positive

*Critical moment* was added to *previous experience*. *Happiness* was deleted as irrelevant. *Motivation* and *awareness* were deleted as these quotes served as examples to justify the importance in mental health (network effects), and therefore, the examples had other codes where network processes were identified. *Sociodemographic factors* and *availability of free time* were added.

Individual negative

*Genre*, *work*, *marital status*, *socioeconomic status* were merged into the new code *sociodemographic factors*. *Self-management* was deleted as irrelevant. *Mindset* and *mental health* were deleted since they were going to be included as part of the narrative (explanation of the story). Therefore, they were not relevant as codes. *Previous experience* was added as a new code.

Processes, ties and outcomes

Negative processes

*Competing*, *diffusion of practices*, *exercising* and *socialising* were deleted as irrelevant. *Social support* was included in *peer support*. The codes *patronising*, *judging*, and *labelling* were merged into the code *judging, bullying and labelling*. *Advising* was included in *counselling and mentoring. Cooking and shopping* were put together, the same as *counselling and mentoring*. *Judging, bullying and labelling*, *cooking and shopping*, *collective efficacy* and *counselling and mentoring* were added as minor codes within the code *peer support*.

Positive processes

*Competing*, *social awareness*, *companion*, *social prescription*, *caring*, *walking* and *trust* were deleted as irrelevant. *Advice* was included in the code *counselling and mentoring*. *Counselling and mentoring* and *cooking and shopping* were created. *Emotional*, *counselling and mentoring*, *collective efficacy* and *cooking and shopping* were included as more minor codes in the code *peer support*.

Template version 2

| **Type of ties**  ~~Online networks~~  ex-husband  Sport contacts and personal trainers  Pet  Neighbours  Sport contacts  Co-workers  Community group  Housemates  Friends  Family  Aunts and uncles  Children  Siblings  Grandparents  Partner  Parents  Healthcare professionals  Classmates  **Contextual and individual factors**  Contextual  Negative  ~~Prices~~  ~~Family socioeconomic status~~  ~~School activities~~  ~~Bars and pubs~~  ~~War times~~  ~~Change country~~  Coronavirus  Working environment  Built environment  Social norms  Social events  Positive  Social norms  Built environment and green  spaces  Social media  School  ~~Networks with conditions~~  ~~New country~~  ~~Community services~~  ~~Incorporation to work~~  Individual  Positive  Knowledge  Availability of free time  Mindset  Sociodemographic factors  Previous experience  Age  ~~Motivation~~  ~~Awareness~~  ~~Critical moment~~  ~~Happiness~~  Negative  ~~Marital status~~  ~~Socioeconomic status~~  ~~Work~~  ~~Genre~~  ~~Self-management~~  ~~Mindset~~  ~~Mental health~~  physical appearance  Previous experience  Sociodemographic factors | **Processes, ties and outcomes**  Negative  ~~Social support~~  ~~Competing~~  ~~Diffusion of practices~~  Isolation  Death  Social modelling  Homophily  ~~Socialising~~  Social comparison  Peer pressure  Peer support  Judging, bullying & labelling  Cooking and shopping  Collective efficacy  Counselling and mentoring  ~~Patronising~~  ~~Judging~~  ~~Exercising~~  ~~Labelling~~  ~~Advising~~  Positive  ~~Competing~~  ~~Social awareness~~  Homophily  Community support  Social modelling  ~~Companion~~  ~~Social prescription~~  ~~Caring~~  ~~Walking~~  ~~Advice~~  ~~Trust~~  Peer support  Emotional  Counselling and mentoring  Collective efficacy  Cooking and shopping  Social comparison  Peer pressure  **Importance of networks**  **Network structure and dynamics of change** |
| --- | --- |

TEMPLATE VERSION 3

From version 2 to 3, there were significant changes. Firstly, *types of ties* and *processes* were merged. Thus, it was decided to establish two themes according to a multilevel analysis based on our previous critical interpretative synthesis review (*Micro-relationships* and *its processes and meso-relationships and its processes*). The *importance of networks* was deleted as a theme. The theme *contextual and individual factors* was modified. Thus, some relevant individual attributes (e.g. *mindset*, *previous experience* or *knowledge*) were added into the different *network processes*. The part regarding *contextual factors* was tagged with the theme *Macro-conditions influence network outcomes* and the *coronavirus* and *socio-cultural conditions* were the broader codes within this category.

Template version 3

| **Macro-conditions influence network outcomes**  Coronavirus  Negative effects  Positive effects  Socio-cultural conditions  Negative effects  Working environment  Built environment  Social norms  Social events  War  Economic  Positive effects  Time  Built environment and green spaces  Social norms  Schools  Social media  **Visualisation of networks**  Structure  Dynamics  **Micro-relationships and its processes**  Family networks  Family (in general)  Negative effects  Homophily  Peer pressure  Social modelling  Peer support  Judging, bullying and labelling  Collective efficacy  Positive effects  Social modelling  Peer pressure  Peer support  Collective efficacy  Counselling and mentoring  Pets  Negative effects  Peer support  Collective efficacy  Positive effects  Peer support  Collective efficacy  Partners  Negative effects  Homophily  Peer pressure  Social comparison  Peer support  Collective efficacy  Positive effects  Peer pressure  Peer support  Cooking and shopping  individual factor previous experience  Counselling and mentoring  Emotional  Collective efficacy  Social comparison  Social modelling  Children  Negative effects  Peer support  Cooking and shopping  Positive effects  Homophily  Peer support  Emotional  Cooking and shopping  Counselling and mentoring  Individual factor_knowledge  Peer pressure  Aunts and uncles  Negative effects  Peer pressure  Grandparents  Negative effects  Peer pressure  Peer support  Judging, bullying and labelling  Cooking and shopping  Positive effects  Social comparison  Individual factor previous experience  Peer support  Emotional  Siblings  Negative effects  Peer pressure  Death  Peer support  Judging, bullying and labelling  Positive effects  Peer support  Emotional  Counselling and mentoring  Parents  Negative effects  Peer pressure  Peer support  Collective efficacy  Judging and labelling  Cooking and shopping  Social modelling  Positive effects  Social modelling  Social comparison  Individual factor mindset  Individual factor previous experience  Peer pressure  Peer support  Collective efficacy  Counselling and mentoring  Emotional  Cooking and shopping  Friends networks  Negative effects  Homophily  Social comparison  Peer support  Judging, bullying and labelling  Cooking and shopping  Collective efficacy  Counselling and mentoring  Peer pressure  Positive effects  Homophily  individual factor mindset  Peer pressure  Social modelling  Social comparison  individual factor previous experience  Peer support  Cooking and shopping  Collective efficacy  Counselling and mentoring  Emotional  **Meso-relationships and its processes**  Community relationships  Classmates  Negative effects  Social comparison  Peer support  Judging, bullying and labelling  Positive effects  Social comparison  Sport contacts and personal trainers  Negative effects  Social comparison  Peer support  Counselling and mentoring  Positive effects  Social comparison  Homophily  Peer pressure  Peer support  Counselling and mentoring  Neighbours  Negative effects  Peer pressure  Positive effects  Social comparison  Co-workers  Negative effects  Peer pressure  Peer support  Collective efficacy  Positive effects  Social modelling  Social comparison  Homophily  Individual factor mindset  Peer support  Emotional  Collective efficacy  Community group  Negative effects  Social comparison  Positive effects  Social modelling  individual factor previous experience  Peer pressure  Homophily  Collective efficacy  Community support  Healthcare professionals  Negative effects  Peer support  Counselling and mentoring  Judging, bullying and labelling  Positive effects  Peer support  Emotional  Counselling and mentoring  individual factor mindset |
| --- |

TEMPLATE VERSION 4

In this version, we removed the theme *visualisation of networks* and everything related to networks' structure and dynamics. After meeting with the rest of the authors, we suggested that there was too much information in the paper and the part related to the structure should be added elsewhere. Also, we ignored the different levels of analysis (meso-micro) to differentiate different types of networks since this could avoid theoretical confusion. Instead, four new main themes were created to identify the types of networks and encompass the different network processes: *become part of the community*, *healthcare professionals* *are another brick in the wall*, *types of influence processes of family networks*, and *friends and the activities by which they affect health*. These themes represent four types of relationships: community groups and other members from the community, healthcare professionals, family and friends.

Template version 4

| **1.****Macro-determinants influence network outcomes**  Coronavirus  Negative effects  Positive effects  Socio-cultural conditions  Negative effects  Working environment  Built environment  Social norms  Social events  War  Economic  Positive effects  Time  Built environment and green spaces  Social norms  Schools  Social media  **2.Become part of the community**  Classmates  Negative effects  Social comparison  Peer support  Judging, bullying and labelling  Positive effects  Social comparison  Sport contacts and personal trainers  Negative effects  Social comparison  Peer support  Counselling and mentoring  Positive effects  Social comparison  Homophily  Peer pressure  Peer support  Counselling and mentoring  Neighbours  Negative effects  Peer pressure  Positive effects  Social comparison  Co-workers  Negative effects  Peer pressure  Peer support  Collective efficacy  Positive effects  Social modelling  Social comparison  Homophily  Individual factor mindset  Peer support  Emotional  Collective efficacy  Community groups and weight management groups  Negative effects  Social comparison  Positive effects  Social modelling  individual factor previous experiences  Peer pressure  Homophily  Community support  Collective efficacy  **3.Healthcare professionals are another brick in the wall**  Negative effects  Peer support  Counselling and mentoring  Judging, bullying and labelling  Positive effects  Peer support  Emotional  Counselling and mentoring  individual factor mindset  **4.Types of influence processes of family networks**  Family (in general)  Negative effects  Homophily  Peer pressure  Social modelling  Peer support  Judging, bullying and labelling  Collective efficacy  Positive effects  Social modelling  Peer pressure  Peer support  Collective efficacy  Counselling and mentoring  Pets  Negative effects  Peer support  Collective efficacy  Positive effects  Peer support  Collective efficacy  Partners  Negative effects  Homophily  Peer pressure  Social comparison  Peer support  Collective efficacy  Positive effects  Peer pressure  Peer support  Cooking and shopping  individual factor previous experience  Counselling and mentoring  Emotional  Collective efficacy  Social comparison  Social modelling  Children  Negative effects  Peer support  Cooking and shopping  Positive effects  Homophily  Peer support  Emotional  Cooking and shopping  Counselling and mentoring  Individual factor_knowledge  Peer pressure  Aunts and uncles  Negative effects  Peer pressure  Grandparents  Negative effects  Peer pressure  Peer support  Judging, bullying and labelling  Cooking and shopping  Positive effects  Social comparison  Individual factor previous experience  Peer support  Emotional  Siblings  Negative effects  Peer pressure  Death  Peer support  Judging, bullying and labelling  Positive effects  Peer support  Emotional  Counselling and mentoring  Parents  Negative effects  Peer pressure  Peer support  Collective efficacy  Judging, bullying and labelling  Cooking and shopping  Social modelling  Positive effects  Social modelling  Social comparison  Individual factor mindset  Individual factor previous experience  Peer pressure  Peer support  Collective efficacy  Counselling and mentoring  Emotional  Cooking and shopping  **5.** **Friends and the activities by which they affect health**  Negative effects  Homophily  Social comparison  Peer support  Judging, bullying and labelling  Cooking and shopping  Collective efficacy  Counselling and mentoring  Peer pressure  Positive effects  Homophily  individual factor mindset  Peer pressure  Social modelling  Social comparison  individual factor previous experience  Peer support  Cooking and shopping  Collective efficacy  Counselling and mentoring  Emotional |
| --- |

TEMPLATE VERSION 5

The codes related with network processes were created in our previous critical review based on a creative and interpretative process. Since we are studying peoples’ narratives, we have decided to change these more theoretical codes into simple words referring to the activities that induce the adoption of different health practices. For example, *collective efficacy* was changed into *conducting activities together* or *social modelling* during the childhood, which was changed into *growing up seeing and modelling bad practices*.

All the community relationships (weak ties) that had a friendship component were included in the second theme to create a distinction in the main manuscript between the influence of close friendship relationships and weak ties friendship relationships. Also, the names of the themes were modified to specify a pattern of meaning rather than a topic summary (the latter is more common in codebook thematic analysis). This further analysis adds meaning to the results. The theme *macro-determinants influence network outcomes* was removed to simplify the template and focus on relationships and their processes. Some broader social and economic factors were reported when writing up some results, but they were not considered a priority for this manuscript.

Template version 5

| **1.Everyday familial routines matter**  Activities with positive effects on health  Family (in general)  Being a role model  Conducting activities together  Counselling  Parents  Being a role model  Physical loss  Peer pressure  Conducting activities together  Counselling  Emotional support  Education in cooking and eating healthy  Pets  Providing physical exercise  Partners  Cooking and shopping healthy food  Counselling  Emotional support  Sharing lifestyle goals and making joint efforts  Comparing  Being a role model  Children  Having similar characteristics  Emotional support  Cooking and shopping healthy food  Education in eating healthy  Peer pressure  Grandparents  Comparing  Emotional support  Siblings  Counselling  Aunts and uncles  Emotional support  Activities with negative effects on health  Family (in general)  Having similar characteristics  Peer pressure  Growing up seeing and modelling bad practices  Judging, labelling and commenting  Conducting activities together  Parents  Providing an excess of control  Conducting activities together  Cooking and shopping  Growing up seeing bad practices  Pets  Encouraging physical activity  Partners  Having similar characteristics  Peer pressure  Comparing  Conducting activities together  Eating and shopping  Children  Education in cooking healthy  Grandparents  Peer pressure  Cooking and providing excess amount of food  Siblings  Peer pressure  Physical loss  Judging, labelling and commenting  **2.** **Chasing healthier lifestyles and modelling and connecting emotionally with friends**  Activities with positive effects on health  Close friends  Having similar characteristics  Modelling  Comparing  Cooking and shopping healthy food  Conducting activities together  Counselling  Emotional support  Community friends (gym and weight management groups)  Leadership and counselling  Sharing agendas and aims  Conducting activities together  Emotional support  Comparing  Modelling  Peer pressure  Colleagues at work  Emotional support  Conducting activities together  Comparing  Activities with negative effects on health  Close friends  Comparing  Cooking and shopping healthy food  Conducting activities together  Counselling and mentoring  Peer pressure  Community friends (gym and weight management groups)  Comparing  Counselling  **3. Healthcare professionals as negative influencers**  Activities with positive effects on health  Emotional support  Counselling  Activities with negative effects on health  Counselling, patronising and not providing person-centred plans  Communication, lack of a sensitive approach |
| --- |

**STAGE 4: FINALISING THE TEMPLATE**

The template was finalised and applied to all the interview transcripts. The researchers considered a final version of the template when it covered all the aspects of the research aim.

**STAGE 5: WRITING UP**

The final phase was writing up, weaving together the analytic narrative and data extracts and contextualising the analysis regarding current literature.

The final framework consisted of three themes, two sub-themes for each theme and 95 codes. The first theme was divided into two sub-themes in the main manuscript since the sections were relatively balanced in terms of length. The part of the text representing the second theme was not divided since the two sub-sections would not be equal in terms of length (although both positive and negative activities were specified in the text). The third theme in the text represents only negative processes since they were the most relevant.
