# Supplementary material for: The influence of social relationships and activities on the health of adults with obesity: A qualitative study
Source: Health Expect. 2022 Jun 24;25(4):1892–903. doi: 10.1111/hex.13540 (PMC9327877; doi:10.1111/hex.13540)
Supplement: Supplementary file 2 — Supplementary information. [file HEX-25--s001.docx]

**Supplementary material 2. Quality criteria. COREQ and a tool for evaluating thematic analysis (TA) manuscripts.**

**Table 1. Consolidated criteria for reporting qualitative studies (COREQ): 32-item checklist (1)**

| No | Item | Guide questions/description | Answer |
| --- | --- | --- | --- |
| **Domain 1: Research team and reflexivity** | | | |
| Personal characteristics | | | |
| 1 | Interview/facilitator | Which author/s conducted the interview or focus group? | The first author conducted all the interviews. |
| 2 | Credentials | What were the researcher's credentials? E.g. PhD, MD | First author: PhD student  The rest of the authors are Emeritus professor and professor. |
| 3 | Occupation | What was their occupation at the time of the study? | First author: PhD student and research assistant.  The rest of the authors as mentioned in question 2. |
| 4 | Gender | Was the researcher male or female? | First author, male. Not specified in the manuscript. |
| 5 | Experience and training | What experience or training did the researcher have? | The researcher had previous training at the faculty (qualitative courses). Not specified in the manuscript. |
| Relationships with participants | | | |
| 6 | Relationship established | Was a relationship established prior to study commencement? | Only a first contact was established to describe the study and look for potential participants. There is no therapeutic relationship between researchers and participants (specified in the main manuscript). |
| 7 | Participant knowledge of the interviewer | What did the participants know about the researcher? e.g. personal goals, reasons for doing the research | A participant information sheet that explained the research in detail was provided before conducting the interviews. |
| 8 | Interviewer characteristics | What characteristics were reported about the interviewer/facilitator? e.g. Bias, assumptions, reasons and interests in the research topic | The interest in the research topic was explained in the introduction of the participant information sheet. Part of that explanation is specified in the introduction section of this article. |
| **Domain 2: study design** | | | |
| Theoretical framework | | | |
| 9 | Methodological orientation and Theory | What methodological orientation was stated to underpin the study? e.g. grounded theory, discourse analysis, ethnography, phenomenology, content analysis | A constructivist epistemology hermeneutic phenomenology. It is explained in the manuscript. |
| Participant selection | | | |
| 10 | Sampling | How were participants selected? e.g. purposive, convenience, consecutive, snowball | Purposive. It is explained in the text. |
| 11 | Method of approach | How were participants approached? e.g. face-to-face, telephone, mail, email | Email, social media and community context. It is explained in the text. |
| 12 | Sample size | How many participants were in the study? | 19. Specified in the text. |
| 13 | Non-participation | How many people refused to participate or dropped out? Reasons? | None of the participants dropped out. However, 44 community groups (long-term conditions and weight management groups at a local and national level-UK) and 17 individuals declined or did not respond to participating in the study. |
| Setting | | | |
| 14 | Setting of data collection | Where was the data collected? e.g. home, clinic, workplace | Data was collected via videoconference and telephone (specified in the manuscript). Most of the participants were at their homes at the time of the interview. |
| 15 | Presence of non-participants | Was anyone else present besides the participants and researchers? | Before the interview, it was recommended to stay in a quiet place to ensure confidentiality. No more people were visualised at the time of the interview. |
| 16 | Description of sample | What are the important characteristics of the sample? e.g. demographic data, date | A sociodemographic questionnaire was used to collect data, and a table was created in the main manuscript specifying the most relevant characteristics for this study. |
| Data collection | | | |
| 17 | Interview guide | Were questions, prompts, guides provided by the authors? Was it pilot tested? | The questions were created by the principal author and delivered by him. Some insights and a brief description of what he was going to ask were provided in advance. The interview was pilot tested with friends and family members of the first author.  The questions regarding social networks were designed based on our previous literature review. The qualitative research group members of the authors’ affiliation and the two first interviewees provided feedback regarding the type and tone of the questions. |
| 18 | Repeat interviews | Were repeat interviews carried out? If yes, how many? | No. All the interviews were carried out once. |
| 19 | Audio/visual recording | Did the research use audio or visual recording to collect the data? | The interviews were audio-recorded. It is explained in the manuscript. |
| 20 | Field notes | Were field notes made during and/or after the interview or focus group? | Field notes were made when the first author re-read and listened again the interviews. |
| 21 | Duration | What was the duration of the interviews or focus group? | Between 30 and 120 minutes. Explained in the text. |
| 22 | Data saturation | Was data saturation discussed? | Data saturation was used. It is specified in the manuscript. |
| 23 | Transcripts returned | Were transcripts returned to participants for comment and/or correction? | We gave the option to return the individual transcripts to the correspondent interviewee as per request. None of the participants requested the transcript. |
| **Domain 3:analysis and findings** | | | |
| Data analysis | | | |
| 24 | Number of data coders | How many data coders coded the data? | The main researcher coded all the data. The rest of the authors checked the name of the domain summary themes and codes, and suggestions were provided to make changes. |
| 25 | Description of the coding tree | Did authors provide a description of the coding tree? | A coding tree is detailed in supplementary material 1, and table 2 in the main manuscript. |
| 26 | Derivation of themes | Were themes identified in advance or derived from the data? | Both. This is explained in methods. |
| 27 | Software | What software, if applicable, was used to manage the data? | NVIVO version 12. |
| 28 | Participant checking | Did participants provide feedback on the findings? | Participant 1 provided feedback about the results (specified in the manuscript). |
| Reporting | | | |
| 29 | Quotations presented | Were participant quotations presented to illustrate the themes / findings? Was each quotation identified? e.g. participant number | Quotations were presented, and participants were anonymised using numbers, e.g. Participant 1. |
| 30 | Data and findings consistent | Was there consistency between the data presented and the findings? | There is consistency between the data presented and the findings. |
| 31 | Clarity of major themes | Were major themes clearly presented in the findings? | 3 themes were presented as subheadings of the results section. |
| 32 | Clarity of minor themes | Is there a description of diverse cases or discussion of minor themes? | Themes were divided in subthemes. Codes were not specified in a special format since we wanted to focus on the story itself. A table with all themes, sub-themes and codes is in the main manuscript. |

**Table 2. A tool for evaluating thematic analysis (TA) manuscripts for publication: Twenty questions to guide assessment of TA research quality (2)**

| Questions | Answers |
| --- | --- |
| *Adequate choice and explanation of methods and methodology* | |
| 1. Do the authors explain why they are using TA, even if only briefly? | It is described in the data analysis section. |
| 2. Do the authors clearly specify and justify which type of TA they are using? | Codebook (template) thematic analysis. It is described in the data analysis section. |
| 3. Is the use and justification of the specific type of TA consistent with the research questions or aims? | The aim has a naturalistic insight since we want to identify and explore in-depth aspects of social relationships. Also, thematic analysis is consistent with a phenomenology approach. |
| 4. Is there a good ‘fit’ between the theoretical and conceptual underpinnings of the research and the specific type of TA (i.e. is there conceptual coherence)? | The theoretical and conceptual underpinnings are based on social network theory, specifically our critical interpretative synthesis review (3). Codebook thematic analysis starts from an a priori template which was created from the results of our review. This is explained in the manuscript and, with more detail, in supplementary material 1. |
| 5. Is there a good ‘fit’ between the methods of data collection and the specific type of TA? | We used individual semi-structured interviews, which fit well with the use of thematic analysis. |
| 6. Is the specified type of TA consistently enacted throughout the paper? | We think so. Also, we have developed supplementary material 1, where we explained all the phases of the specific analysis step by step. |
| 7. Is there evidence of problematic assumptions about, and practices around, TA? These commonly include:  ● Treating TA as one, homogenous, entity, with one set of – widely agreed on – procedures.  ● Combining philosophically and procedurally incompatible approaches to TA without any acknowledgement or explanation.  ● Confusing summaries of data topics with thematic patterns of shared meaning, underpinned by a core concept.  ● Assuming grounded theory concepts and procedures (e.g. saturation, constant comparative analysis, line-by-line coding) apply to TA without any explanation or justification.  ● Assuming TA is essentialist or realist, or atheoretical.  ● Assuming TA is only a data reduction or descriptive approach and therefore must be supplemented with other methods and procedures to achieve other ends. | All these points were taken into account.  -TA was not treated as one since we are aware that there are different types of TA.  - We use a constructivist epistemology hermeneutic phenomenology methodology to study lived experience. It is consistent with TA.  -We have created three themes. We are aware of the differences.  We know that grounded theory (as an analytical method) is completely different from thematic analysis.  -We are aware that TA is not atheoretical. TA is suited to both experiential (e.g. critical realist, contextualist) and critical (e.g. relativist, constructionist) framings of language, data and meaning.  -TA is not only a data reduction or descriptive approach. There is an interpretative activity conducted by the researchers  through the lenses of their particular social context. Also, we add our opinions during the analytical process. |
| 8. Are any supplementary procedures or methods justified, and necessary, or could the same results have been achieved simply by using TA more effectively? | Not applicable. |
| 9. Are the theoretical underpinnings of the use of TA clearly specified (e.g. ontological, epistemological assumptions, guiding theoretical framework(s)), even when using TA inductively (inductive TA does not equate to analysis in a theoretical vacuum)? | They are explained in the introduction and methodology sections. |
| 10. Do the researchers strive to ‘own their perspectives’ (even if only very briefly), their personal and social standpoint and positioning? (This is especially important when the researchers are engaged in social justice oriented research and when representing the ‘voices’ of marginal and vulnerable groups, and groups to which the researcher does not belong.) | As part of a hermeneutical phenomenology, our opinions as researchers were crucial for the analysis and created the codes and themes. On the other hand, we adjusted as much as possible to the participants’ narratives, so that we did not lose the essence of the lived experience. |
| 11. Are the analytic procedures used clearly outlined, and described in terms of what the authors actually did, rather than generic procedures? | We specified the different steps in supplementary material 1. |
| 12. Is there evidence of conceptual and procedural confusion? For example, reflexive TA (e.g. Braun and Clarke 2006) is the claimed approach but different procedures are outlined such as the use of a codebook or coding frame, multiple independent coders and consensus coding, inter-rater reliability measures, and/or themes are conceptualised as analytic inputs rather than outputs and therefore the analysis progresses from theme identification to coding (rather than coding to theme development). | We have followed the procedure of codebook thematic analysis, although it is true that there are elements of reflexive TA; for example, main themes are not domain summary themes, but they specify a pattern of meaning. We think thematic analysis is flexible enough to conduct this practice, and the article benefits from it. |
| 13. Do the authors demonstrate full and coherent understanding of their claimed approach to TA? | We have justified the use of TA in regard to our aims, philosophy and methodology. |
| A well-developed and justified analysis | |
| 14. Is it clear what and where the themes are in the report? Would the manuscript benefit from some kind of overview of the analysis: listing of themes, narrative overview, table of themes, thematic map? | The themes are subheadings in the results section. A table with a list of final themes and codes is in the main manuscript and more information is provided in supplementary material 1. |
| 15. Are the reported themes topic summaries, rather than ‘fully realised themes’ – patterns of shared meaning underpinned by a central organising concept?  ● If so, are topic summaries appropriate to the purpose of the research?  ○ If the authors are using reflexive TA, is this modification in the conceptualisation of themes explained and justified?  ● Have the data collection questions been used as themes?  ● Would the manuscript benefit from further analysis being undertaken, with the reporting of fully realised themes?  ● Or, if the authors are claiming to use reflexive TA, would the manuscript benefit from claiming to use a different type of TA (e.g. coding reliability or codebook)? | Although we have used the process of codebook thematic analysis, we have developed fully realised themes eventually since we think the manuscript benefits from it. We have not explained (in the main manuscript) this modification in the conceptualisation of the manuscript's themes since there is no more space for it. More details are explained in supplementary material 1. |
| 16. Is non-thematic contextualising information presented as a theme? (e.g. the first 'theme' is a topic summary providing contextualising information, but the rest of the themes reported are fully realised themes). If so, would the manuscript benefit from this being presented as non-thematic contextualising information? | All of them are fully themes. |
| 17. In applied research, do the reported themes have the potential to give rise to actionable outcomes? | In the discussion section, we explained how some of the results could be useful for future interventions. |
| 18. Are there conceptual clashes and confusion in the paper? (e.g. claiming a social constructionist approach while also expressing concern for positivist notions of coding reliability, or claiming a constructionist approach while treating participants’ language as a transparent reflection of their experiences and behaviours) | We follow a constructivist epistemology. We think there are no conceptual clashes in the paper. |
| 19. Is there evidence of weak or unconvincing analysis, such as:  ● Too many or two few themes?  ● Too many theme levels?  ● Confusion between codes and themes?  ● Mismatch between data extracts and analytic claims?  ● Too few or too many data extracts?  ● Overlap between themes? | Three themes were created for the main manuscript.  Codes were differentiated from the wider categories. Also, there are two sub-themes in the first theme. Supplementary material 1 reports the full list of codes, sub-themes and themes. Although each theme has two-sub themes, we have decided to represent only sub-themes in the first theme as they were the most relevant and keep a balance in the main manuscript regarding the length of the sections.  We think that data extracts relate to the created codes.  We have tried to balance the most representative results, a variety of positive and negative effects on health and the number of examples provided to describe each code.  Themes represent different types of networks, so overlapping should have been avoided. |
| 20. Do authors make problematic statements about the lack of generalisability of their results, and or implicitly conceptualise generalisability as statistical probabilistic generalisability | Our results cannot be generalised. This information belongs to individuals who have different stories and similar socio-demographic characteristics. However, the results might provide an idea of the importance of social networks in the obesity and long-term conditions field and a way to explore in-depth and raise awareness of relationships. |

**References**

1. Tong A, Sainsbury P, Craig J. Consolidated criteria for reporting qualitative research (COREQ): a 32-item checklist for interviews and focus groups. Int J Qual Health Care. 2007 Dec 1;19(6):349–57.

2. Braun V, Clarke V. One size fits all? What counts as quality practice in (reflexive) thematic analysis? Qual Res Psychol. 2021 Jul 3;18(3):328–52.

3. Serrano Fuentes N, Rogers A, Portillo MC. Social network influences and the adoption of obesity-related behaviours in adults: a critical interpretative synthesis review. BMC Public Health. 2019 Aug 28;19(1):1178.
